# Supplementary material for: Effects of breathing exercise and thoracic techniques on pain and disability in low back pain: A systematic review and meta-analysis
Source: PLoS One. 2026 Jan 14;21(1):e0339263. doi: 10.1371/journal.pone.0339263 (PMC12803473; doi:10.1371/journal.pone.0339263)
Supplement: S2 Appendix — (DOCX) [file pone.0339263.s002.docx]

**S1 Apendix.** Search syntax:

**Pubmed:**

((lbp[tiab] OR ("low back pain"[tiab]) OR lumbago[tiab] OR ("lower back pain" [tiab]) OR ("low back ache"[tiab]) OR ("low backache"[tiab]) OR ("back pain"[tiab]) OR backache[tiab] OR ("back ache"[tiab]) OR backache[tiab] OR backaches[tiab] OR ("back aches"[tiab]) OR ("back pains" [tiab]) OR ("acute back pain"[tiab]) OR ("recurrent low back pain" [tiab]) OR ("chronic low back pain"[tiab]) OR ("non-specific low back pain" [tiab]) OR ("low backache"[tiab]) OR ("low back ache"[tiab])) AND ((diaphragm exercise) OR (respiratory diaphragm exercise) OR (diaphragm, respiratory exercise) OR (respiratory diaphragm exercise) OR (respiratory exercise) OR (pulmonary exercise) OR (lung exercise) OR (respiratory rehabilitation) OR (pulmonary rehabilitation) OR (lung rehabilitation) OR (diaphragm breathing) OR (diaphragm strengthen) OR (diaphragm resistance) OR (diaphragm releasing) OR (breathing exercise) OR (diaphragm*) OR (breath*) OR (inspiratory muscle training) OR (respiratory muscle) OR (respiratory muscle strength) OR (inspiration) OR (thoracic osteopathic manipulat*) OR (thorax osteopathic manipulat*) OR (trunk osteopathic manipulat*) OR (thoracic chiropractic manipulat*) OR (thorax chiropractic manipulat*) OR (trunk chiropractic manipulat*) OR (thoracic adjust*) OR (thorax adjust*) OR (trunk adjust*) OR (thoracic chiropractic) OR (trunk chiropractic) OR (thorax chiropractic) OR (chiropractic thoracic adjust*) OR (chiropractic trunk adjust*) OR (chiropractic thorax adjust*) OR (thoracic manipulat*) OR (trunk manipulat*) OR (thorax manipulat*) OR (thoracic manual therap*) OR (thorax manual therap*) OR (trunk manual therap*) OR (thoracic AND release) OR (trunk AND release) OR (thorax AND release) OR (thoracic mobilization))

**Scopus:**

((TITLE-ABS(lbp) OR TITLE-ABS("low back pain") OR TITLE-ABS(Lumbago) OR TITLE-ABS("Lower Back Pain") OR TITLE-ABS("Low Back Ache") OR TITLE-ABS("Low Backache") OR TITLE-ABS("Back pain") OR TITLE-ABS(Backache) OR TITLE-ABS("Back Ache") OR TITLE-ABS(Backache) OR TITLE-ABS( Backaches) OR TITLE-ABS("Back Ache") OR TITLE-ABS("Back Aches") OR TITLE-ABS("Back Pains") OR TITLE-ABS("acute back pain") OR TITLE-ABS("recurrent low back pain") OR TITLE-ABS("chronic low back pain") OR TITLE-ABS("non-specific low back pain") OR TITLE-ABS("low backache") OR TITLE-ABS("low back ache")) AND (ALL("Diaphragm exercise") OR ALL("Respiratory Diaphragm exercise") OR (ALL(Diaphragm) AND ALL("Respiratory exercise")) OR ALL("respiratory exercise") OR ALL("pulmonary exercise") OR ALL("lung exercise") OR ALL("respiratory rehabilitation") OR ALL("pulmonary rehabilitation") OR ALL("lung rehabilitation") OR ALL("diaphragm breathing") OR ALL("diaphragm strengthen") OR ALL("diaphragm resistance") OR ALL("diaphragm releasing") OR ALL("breathing exercise") OR TITLE-ABS("diaphragm*") OR TITLE-ABS("breath*") OR ALL("inspiratory muscle training") OR ALL("respiratory muscle") OR ALL("respiratory muscle strength") OR ALL(thoracic osteopathic manipulat*) OR ALL(thorax osteopathic manipulat*) OR ALL(trunk osteopathic manipulat*) OR ALL(thoracic chiropractic manipulat*) OR ALL(thorax chiropractic manipulat*) OR ALL(trunk chiropractic manipulat*) OR ALL(thoracic adjust*) OR ALL(thorax adjust*) OR ALL(trunk adjust*) OR ALL(thoracic chiropractic) OR ALL(trunk chiropractic) OR ALL(thorax chiropractic) OR ALL(chiropractic thoracic adjust*) OR ALL(chiropractic trunk adjust*) OR ALL(chiropractic thorax adjust*) OR ALL(thoracic manipulat*) OR ALL(trunk manipulat*) OR ALL(thorax manipulat*) OR ALL(thoracic manual therap*) OR ALL(thorax manual therap*) OR ALL(trunk manual therap*) OR (ALL(thoracic) AND ALL(release)) OR (ALL(trunk) AND ALL(release)) OR (ALL(thorax) AND ALL(release)) OR (thoracic mobil*))

**WOS:**

((TS=(lbp) OR TS=("low back pain") OR TS=(Lumbago) OR TS=("Lower Back Pain") OR TS=("Low Back Ache") OR TS=("Low Backache") OR TS=("Back pain") OR TS=(Backache) OR TS=("Back Ache") OR TS=(Backache) OR TS=( Backaches) OR TS=("Back Ache") OR TS=("Back Aches") OR TS=("Back Pains") OR TS=("acute back pain") OR TS=("recurrent low back pain") OR TS=("chronic low back pain") OR TS=("non-specific low back pain") OR TS=("low backache") OR TS=("low back ache")) AND (ALL=("Diaphragm exercise") OR ALL=("Respiratory Diaphragm exercise") OR (ALL=(Diaphragm) AND ALL=("Respiratory exercise")) OR ALL=("respiratory exercise") OR ALL=("pulmonary exercise") OR ALL=("lung exercise") OR ALL=("respiratory rehabilitation") OR ALL=("pulmonary rehabilitation") OR ALL=("lung rehabilitation") OR ALL=("diaphragm breathing") OR ALL= ("diaphragm strengthen") OR ALL=("diaphragm resistance") OR ALL=("diaphragm releasing") OR ALL=("breathing exercise") OR TS=("diaphragm*") OR TS=("breath*") OR ALL=("inspiratory muscle training") OR ALL=("respiratory muscle") OR ALL=("respiratory muscle strength") OR ALL=(thoracic osteopathic manipulat*) OR ALL=(thorax osteopathic manipulat*) OR ALL=(trunk osteopathic manipulat*) OR ALL=(thoracic chiropractic manipulat*) OR ALL=(thorax chiropractic manipulat*) OR ALL=(trunk chiropractic manipulat*) OR ALL=(thoracic adjust*) OR ALL=(thorax adjust*) OR ALL=(trunk adjust*) OR ALL=(thoracic chiropractic) OR ALL=(trunk chiropractic) OR ALL=(thorax chiropractic) OR ALL=(chiropractic thoracic adjust*) OR (ALL=(chiropractic trunk adjust*) OR ALL=(chiropractic thorax adjust*) OR ALL=(thoracic manipulat*) OR ALL=(trunk manipulat*) OR ALL=(thorax manipulat*) OR ALL=(thoracic manual therap*) OR ALL=(thorax manual therap*) OR ALL=(trunk manual therap*) OR ALL=(thoracic Mobil*))

**Ovid:**

(lbp.ab. or "low back pain".ab. or lumbago.ab. or "lower back pain".ab. or "low back ache".ab. or "low backache".ab. or "back pain".ab. or backache.ab. or "back ache".ab. or backache.ab. or backaches.ab. or "back aches".ab. or "back pains".ab. or "acute back pain".ab. or "recurrent low back pain".ab. or "chronic low back pain".ab. or "non-specific low back pain".ab. or "low backache".ab. or "low back ache".ab.) and ("diaphragm exercise".af. or "respiratory diaphragm exercise".af. or (diaphragm.af. and "respiratory exercise".af.) or "respiratory diaphragm exercise".af. or "respiratory exercise".af. or "pulmonary exercise".af. or "lung exercise".af. or "respiratory rehabilitation".af. or "pulmonary rehabilitation".af. or "lung rehabilitation".af. or diaphragm*.af. or breath*.af. or "inspiratory muscle training".af. or "respiratory muscle".af. or "respiratory muscle strength".af. or "inspiration".af. or "thoracic osteopathic manipulat*".af. or "thorax osteopathic manipulat*".af. or "trunk osteopathic manipulat*".af. or "thoracic chiropractic manipulat*".af. or "thorax chiropractic manipulat*".af. or "trunk chiropractic manipulat*".af. or "thoracic adjust*".af. or "thorax adjust*".af. or "trunk adjust*".af. or "thoracic chiropractic".af. or "trunk chiropractic".af. or "thorax chiropractic".af. or "chiropractic thoracic adjust*".af. or "chiropractic trunk adjust*".af. or "chiropractic thorax adjust*".af. or "thoracic manipulat*".af. or "trunk manipulat*".af. or "thorax manipulat*".af. or "thoracic manual therap*".af. or "thorax manual therap*".af. or "trunk manual therap*".af. or (thoracic and release).af. or (trunk and release).af or (thorax and release).af. or (thoracic mobil*).af.)

**CENTRAL:**

(lbp OR ("low back pain") OR lumbago OR ("lower back pain") OR ("low back ache") OR ("low backache") OR ("back pain") OR backache OR ("back ache") OR backache OR backaches OR ("back aches") OR ("back pains") OR ("acute back pain") OR ("recurrent low back pain") OR ("chronic low back pain") OR ("non-specific low back pain") OR ("low backache") OR ("low back ache")) AND ((diaphragm exercise) OR (respiratory diaphragm exercise) OR (diaphragm, respiratory exercise) OR (respiratory diaphragm exercise) OR (respiratory exercise) OR (pulmonary exercise) OR (lung exercise) OR (respiratory rehabilitation) OR (pulmonary rehabilitation) OR (lung rehabilitation) OR (diaphragm breathing) OR (diaphragm strengthen) OR (diaphragm resistance) OR (diaphragm releasing) OR (breathing exercise) OR (diaphragm*) OR (breath*) OR (inspiratory muscle training) OR (respiratory muscle) OR (respiratory muscle strength) OR (inspiration) OR (thoracic osteopathic manipulat*) OR (thorax osteopathic manipulat*) OR (trunk osteopathic manipulat*) OR (thoracic chiropractic manipulat*) OR (thorax chiropractic manipulat*) OR (trunk chiropractic manipulat*) OR (thoracic adjust*) OR (thorax adjust*) OR (trunk adjust*) OR (thoracic chiropractic) OR (trunk chiropractic) OR (thorax chiropractic) OR (chiropractic thoracic adjust*) OR (chiropractic trunk adjust*) OR (chiropractic thorax adjust*) OR (thoracic manipulat*) OR (trunk manipulat*) OR (thorax manipulat*) OR (thoracic manual therap*) OR (thorax manual therap*) OR (trunk manual therap*) OR (thoracic AND release) OR (trunk AND release) OR (thorax AND release))

**ProQuest:**

((ABSTRACT,TITLE(lbp) OR ABSTRACT,TITLE("low back pain") OR ABSTRACT,TITLE(Lumbago) OR ABSTRACT,TITLE("Lower Back Pain") OR ABSTRACT,TITLE("Low Back Ache") OR ABSTRACT,TITLE("Low Backache") OR ABSTRACT,TITLE("Back pain") OR ABSTRACT,TITLE(Backache) OR ABSTRACT,TITLE("Back Ache") OR ABSTRACT,TITLE(Backache) OR ABSTRACT,TITLE( Backaches) OR ABSTRACT,TITLE("Back Ache") OR ABSTRACT,TITLE("Back Aches") OR ABSTRACT,TITLE("Back Pains") OR ABSTRACT,TITLE("acute back pain") OR ABSTRACT,TITLE("recurrent low back pain") OR ABSTRACT,TITLE("chronic low back pain") OR ABSTRACT,TITLE("non-specific low back pain") OR ABSTRACT,TITLE("low backache") OR ABSTRACT,TITLE("low back ache")) AND (FULLTEXT("Diaphragm exercise") OR FULLTEXT("Respiratory Diaphragm exercise") OR (FULLTEXT(Diaphragm) AND FULLTEXT("Respiratory exercise")) OR FULLTEXT("respiratory exercise") OR FULLTEXT("pulmonary exercise") OR FULLTEXT("lung exercise") OR FULLTEXT("respiratory rehabilitation") OR FULLTEXT("pulmonary rehabilitation") OR FULLTEXT("lung rehabilitation") OR FULLTEXT("diaphragm breathing") OR FULLTEXT("diaphragm strengthen") OR FULLTEXT("diaphragm resistance") OR FULLTEXT("diaphragm releasing") OR FULLTEXT("breathing exercise") OR ABSTRACT,TITLE("diaphragm*") OR ABSTRACT,TITLE("breath*") OR FULLTEXT("inspiratory muscle training") OR FULLTEXT("respiratory muscle") OR FULLTEXT("respiratory muscle strength") OR FULLTEXT(thoracic osteopathic manipulat*) OR FULLTEXT(thorax osteopathic manipulat*) OR FULLTEXT(trunk osteopathic manipulat*) OR FULLTEXT(thoracic chiropractic manipulat*) OR FULLTEXT(thorax chiropractic manipulat*) OR FULLTEXT(trunk chiropractic manipulat*) OR FULLTEXT(thoracic adjust*) OR FULLTEXT(thorax adjust*) OR FULLTEXT(trunk adjust*) OR FULLTEXT(thoracic chiropractic) OR FULLTEXT(trunk chiropractic) OR FULLTEXT(thorax chiropractic) OR FULLTEXT(chiropractic thoracic adjust*) OR FULLTEXT(chiropractic trunk adjust*) OR FULLTEXT(chiropractic thorax adjust*) OR FULLTEXT(thoracic manipulat*) OR FULLTEXT(trunk manipulat*) OR FULLTEXT(thorax manipulat*) OR FULLTEXT(thoracic manual therap*) OR FULLTEXT(thorax manual therap*) OR FULLTEXT(trunk manual therap*) OR (FULLTEXT(thoracic) AND FULLTEXT(release)) OR (FULLTEXT(trunk) AND FULLTEXT(release)) OR (FULLTEXT(thorax) AND FULLTEXT(release)))
