# Supplementary material for: Effects of breathing exercise and thoracic techniques on pain and disability in low back pain: A systematic review and meta-analysis
Source: PLoS One. 2026 Jan 14;21(1):e0339263. doi: 10.1371/journal.pone.0339263 (PMC12803473; doi:10.1371/journal.pone.0339263)
Supplement: Attachment — Submitted filename: Responds to the comments made on the original manuscript.docx [file pone.0339263.s004.docx]

Dear Respected Editor,

This study is a revised version of a previous manuscript submitted to Plos One with the submission number PONE-D-25-23018, which was rejected due to the editor’s concerns about updating the research. Dr. Jasmine Gomes's email can be found following this cover letter. The search period has been updated to May 1, 2025, and nine studies have been added to the meta-analysis. All analyses and results have been updated accordingly.

Your sincerely,

Tahere Seyedhoseinpoor

Assistant professor, Mobility Impairment Research Center, Health Institute, Babol University of Medical Sciences, Babol, Iran.

[Tahere.hoseinpoor.65@gmail.com](mailto:Tahere.hoseinpoor.65@gmail.com)

Dr. Jasmine Gomes's email:

PONE-D-25-23018
Effects of Breathing Exercise and Thoracic Techniques on Pain and Disability in Low Back Pain: A Systematic Review and Meta-analysis
PLOS ONE

Dear Dr. Seyedhoseinpoor,

Thank you for submitting your manuscript for review by PLOS ONE. After careful consideration, we have decided that your manuscript does not meet our standards for systematic and scoping reviews. We are therefore rejecting your manuscript.

PLOS ONE considers systematic reviews, scoping reviews and meta-analyses for publication; however, these submissions must address a clear and defined research question; conduct a systematic and comprehensive literature review; and use clearly reported, reproducible, and systematic methods to identify, select, and critically appraise relevant research. Our author guidelines for systematic reviews are at [https://journals.plos.org/plosone/s/submission-guidelines#loc-systematic-reviews-and-meta-analyses](https://track.editorialmanager.com/CL0/https:%2F%2Fjournals.plos.org%2Fplosone%2Fs%2Fsubmission-guidelines%23loc-systematic-reviews-and-meta-analyses/1/010f01968a7c93a0-05c0ac96-b7e0-4f16-9f96-c0209e6a23ee-000000/L0uomJo46JpkF5-9qVFlGJfzjBiNXLtptyhuird8Nk8=208).

In the case of your submission, we are concerned that the literature review was completed over a year ago. Systematic and scoping reviews should provide an up-to-date overview of the research topic covered, and we do not feel that this study meets this requirement.

As a result of these concerns, we cannot consider the manuscript for publication at PLOS ONE at this time. However, if you are able to address the concerns discussed above, we may be able to consider a revised version of the manuscript. This would however require a substantial amount of work and thus, would be considered as a new submission. If you are able to revise the manuscript to respond to the concerns noted above, please refer to the original submission in the covering letter and accompany the submission by a rebuttal letter that responds to the comments made on the original manuscript.

I am sorry that we cannot be more positive on this occasion, but I hope you understand the reasons for this decision.

Yours sincerely,

Jasmine Gomes, Ph.D

Editorial Office

PLOS ONE.
