# Supplementary material for: Effects of breathing exercise and thoracic techniques on pain and disability in low back pain: A systematic review and meta-analysis
Source: PLoS One. 2026 Jan 14;21(1):e0339263. doi: 10.1371/journal.pone.0339263 (PMC12803473; doi:10.1371/journal.pone.0339263)
Supplement: Attachment — Submitted filename: Response to Reviewers.docx [file pone.0339263.s005.docx]

**Dear Editor and Reviewers,**

We sincerely thank you for your thoughtful and constructive comments, which have greatly helped us improve the quality of our manuscript. Below, we provide detailed, point-by-point responses to each reviewer’s comments.

With best regards

| ***Reviewer 1*** | | |
| --- | --- | --- |
| ***Comments*** | | ***Reply*** |
| 1 | I recommend adding a few sentences in the introduction, about the effectiveness and usefulness of the Effects of Breathing Exercise in various musculoskeletal disorders, neck pain, shoulder pain, low back pain. | Thank you for this valuable suggestion. We have revised the Introduction to include additional evidence on the effectiveness of breathing exercises in musculoskeletal disorders beyond low back pain. Specifically, we now highlight studies demonstrating that breathing reeducation can reduce pain and improve cervical function in chronic neck pain, enhance mobility and quality of life in shoulder dysfunction, and contribute to the correction of postural abnormalities such as thoracic kyphosis and forward head posture. These additions strengthen the rationale for systematically investigating the role of breathing exercises in CLBP.  The following text was added to the Introduction (page 4, paragraph 4):  **“Supporting evidence from other musculoskeletal conditions further strengthens the rationale for this work. Several studies have demonstrated that breathing exercises improve pain, function, and quality of life in chronic neck pain, shoulder dysfunction, and postural syndromes such as thoracic kyphosis and forward head posture. These findings suggest that breathing interventions exert musculoskeletal benefits beyond LBP, likely through their influence on neuromuscular coordination, posture, and pain modulation.”**  In addition, minor revisions were made in paragraph 5 to improve clarity and flow. |
| 2 | Discussions should be reviewed in light of the overall improvement of the paper. Redundant sentences and prewritten information should be avoided. Focus on take-home messages and how that information impacts the clinical practice of management these patients. | Thank you for this insightful comment. In line with your suggestion, we have revised the *Discussion* section to minimize redundancy and remove prewritten background material. The revised version now places greater emphasis on the key findings and their clinical implications, thereby improving clarity, conciseness, and relevance to clinical practice. All modifications have been clearly marked using *track changes* for transparency. |
| 3 | I suggest to add this reference: Cefalì A, Santini D, Lopez G, Maselli F, Rossettini G, Crestani M, Lullo G, Young I, Dunning J, de Abreu RM, Mourad F. Effects of Breathing Exercises on Neck Pain Management: A Systematic Review with Meta-Analysis. J Clin Med. 2025 Jan 22;14(3). doi: 10.3390/jcm14030709. Review. PubMed PMID: 39941380; PubMed Central PMCID: PMC11818914. | Thank you for this helpful suggestion. We have incorporated the recommended reference (Cefalì et al., 2025) into the Introduction to strengthen the evidence supporting the effectiveness of breathing exercises in musculoskeletal disorders beyond low back pain. This work has been cited as reference **21** in the revised manuscript. |

| ***Reviewer 2*** | | |
| --- | --- | --- |
| ***Major Comments*** | | ***Reply*** |
| 1 | Heterogeneity: High heterogeneity (I² > 85% for pain, >90% for disability) persists despite subgroup analyses. While some reductions occur (e.g., I² = 30.5% for >4 weeks treatment in disability), sources remain unclear. Discuss potential unmeasured factors (e.g., LBP chronicity, co-interventions, outcome measurement timing) more deeply.  Consider metaregression if data allow, or explicitly state why it was not performed.  In the revised version, the shift to SMD in some sections (e.g., page 67) is inconsistent with Morris' dppc—clarify and standardize. | Thank you for this important comment. We agree that the high heterogeneity requires deeper consideration. In the revised manuscript, we now discuss additional potential unmeasured factors that may have contributed to the heterogeneity, including **differences in LBP chronicity, co-interventions, treatment duration, exercise intensity, and timing of outcome assessments**. Notably, subgroup analysis showed that treatment duration longer than four weeks reduced heterogeneity for disability outcomes to approximately 30%, suggesting its role as a key moderator. These points are now discussed in **Discussion (paragraph 5)**, highlighted in the **Limitations section**, and reiterated in the **second sentence of the Conclusion** to emphasize their implications for interpreting and applying the findings.  Thank you for your comment. We chose **subgroup analysis** over meta-regression for the following reasons: Many of the moderators of interest (treatment type, patient and rater blinding, risk of bias) were categorical, and the total number of included studies (24 and 31 in the separate meta-analyses) was not sufficient to support a robust meta-regression with multiple moderators. Subgroup analysis is more appropriate in this context and aligns with PRISMA and Cochrane recommendations for meta-analyses with a modest number of studies. These points are clarified in the **Methods sections (2^nd^ paragraph of the Assessment and Investigation of Heterogeneity part)** of the manuscript.  Thank you for your comment. In the updated version of the manuscript, we consistently used **Morris’s delta (dppc)** instead of SMD to estimate the magnitude of the treatment effect, as this method accounts for pre–post differences in both treatment and control groups—a limitation of using SMD in this context. The reference on page 67 to SMD was only present in deleted tracked changes and does not appear in the current version. Therefore, all effect sizes are now standardized using Morris’s delta throughout the manuscript for consistency. |
| 2 | Quality of Evidence and Interpretation: GRADE rates evidence as low/very low, yet conclusions emphasize effectiveness (e.g., "thoracic techniques are more effective"). Temper claims to reflect this (e.g., "may be effective based on low-quality evidence"). Highlight implications for practice: given low evidence, these interventions should be adjunctive, not first-line. Address why thoracic techniques show larger effects— is it due to higher bias in those studies? | Thank you for your valuable comment. We have revised the manuscript to temper claims about effectiveness in line with the low GRADE ratings. Phrases emphasizing results now reflect caution (e.g., “indicating caution in interpreting the results,” “may provide superior outcomes”). We explicitly addressed that thoracic techniques showed larger effects potentially due to bias in some studies. Implications for practice are clarified, emphasizing that these interventions should be considered adjunctive rather than first-line. These revisions are reflected in **Discussion, the Strengths and Limitations section, and the Conclusion**. |
| 3 | Publication Bias: Funnel plots suggest bias for disability (and pain in some versions), but Egger's/trim-and-fill do not. This discrepancy warrants discussion. The jackknife method identifies one influential study (Ho-Hee 2015) for disability— exclude it in sensitivity analysis and report if results change substantially | Thank you for this comment. We have discussed the assessment of publication bias and sensitivity analysis in the revised manuscript. **Egger’s regression and the trim-and-fill method did not indicate substantial publication bias**, but funnel plots suggested potential bias for both pain intensity and disability outcomes. **Sensitivity analysis using the leave-one-out method showed that most studies did not significantly affect the pooled estimates, except for the Ho-Hee (29) study, which influenced the pooled disability effect size.** These findings are now addressed in the Discussion section (last paragraph before strengths and limitations section), highlighting the robustness of our results while acknowledging uncertainty due to possible publication bias and individual study influence. |
| 4 | Search Update: The search ends May 1, 2025, but the current date is September 24, 2025. While the revision addresses prior PLOS ONE concerns, perform a quick update to ensure no new studies (e.g., via PubMed alert). If none, state this. The inclusion of non-English studies (Korean, Chinese, Spanish) is a strength, but confirm translations were accurate. | We appreciate the reviewer’s insightful comment. The original literature search was completed on **May 1, 2025**, and the current submission date is **September 24, 2025**, which falls within an acceptable six-month period for systematic reviews, as recommended in methodological guidelines for timely updates. A brief verification using **PubMed alerts** was conducted to identify any newly published studies since the original search, and **no additional eligible studies were identified**. Furthermore, we confirm that all **non-English studies (Korean, Chinese, Spanish)** included in the review were accurately translated, ensuring the integrity and reliability of the extracted data. (mentioned in the last sentence of the first paragraph of the **Result** section.) |
| 5 | Outcome Measures: Pain and disability are primary, but tools vary (VAS/NRS for pain, ODI/RMQ for disability). Discuss if this contributes to heterogeneity. Consider pooled results by specific tool in subgroups. Also, the abstract reports 36 studies in meta-analysis, but text specifies 31 for pain/24 for disability—clarify. | We thank the reviewer for this important comment.  We acknowledge that **variation in outcome measurement tools** (e.g., VAS/NRS for pain, ODI/RMQ for disability) may have contributed to heterogeneity. To account for these differences, **Morris’s delta was used as a standardized effect size**, allowing us to estimate treatment effects while considering pre–post differences in both intervention and control groups. However, due to these differences in scales, **we were unable to assess minimal clinically important differences (MCID)**, which we now explicitly acknowledge as a limitation.  As described in the Methods section, **low back pain intensity and disability were predefined as the primary outcomes**, and included studies were required to report at least one of these outcomes. Among the 36 studies included in the review, **31 reported pain outcomes** and **24 reported disability outcomes**, with some studies reporting only pain or only disability. This has now been mentioned at the beginning of the second paragraph of the **Result section.** And the abstract has been revised. |
| ***Minor Comments*** | | ***Reply*** |
| 6 | Abstract: Update to reflect revised effect sizes (e.g., large for disability). Specify "thoracic manual techniques" for clarity. - | We thank the reviewer for these helpful suggestions. In line with the comments, we have revised the Abstract to:   1. Reflect the updated effect sizes, explicitly noting a **large effect for disability** and a **small effect for pain**. And some revision on conclusion focusing on uncertainity of results. 2. Specify “**thoracic manual techniques**” for clarity, with examples provided (mobilization, mobilization, high-velocity low-amplitude manipulation, and release techniques).   The revised Abstract now more accurately conveys the magnitude of the findings and the scope of included interventions. |
| 7 | Methods: Search syntax in S1 Appendix is noted—ensure it's included. Define "thoracic techniques" more precisely (e.g., HVLA vs. mobilization). | Thank you for this valuable comment. We have ensured that the full **search syntax is provided in S1 Appendix** for transparency and reproducibility. In addition, the term **“thoracic techniques” has been clarified in the Methods section** to specify that it includes thoracic manual techniques such as high-velocity low-amplitude (HVLA) manipulation, mobilization, and release techniques. |
| 8 | Results: Tables 2-4 are clear, but label effect sizes consistently (Morris' dppc vs. SMD in revisions). Figures 3-7 need legends for clarity. | Thank you for this important observation. We confirm that the effect size is consistently reported as **Morris’ dppc** throughout the manuscript. References to SMD appeared only in earlier tracked revisions and have now been fully removed to maintain consistency. Furthermore, **legends for Figures 3–7 have been added and formatted according to the journal’s author guidelines within the manuscript**, ensuring clarity and alignment with reporting standards. |
| 9 | Discussion: Integrate recent studies (added in revision) more explicitly. Compare to Fabero-Garrido et al. (2024) on respiratory muscle training. | Thank you for this valuable suggestion. We have revised the Discussion (1^st^ paragraph) to explicitly integrate recent systematic reviews and meta-analyses, including **Jiang et al. (2024), Shi et al. (2023), Zhai et al. (2024),** and **Fabero-Garrido et al. (2024)**. In particular, we compare our findings to **Fabero-Garrido et al.,** highlighting that respiratory muscle training improves pain, disability, and respiratory function, which complements our results on thoracic and breathing interventions. These additions clarify how our findings align with and extend the most recent evidence, emphasizing the clinical relevance of respiratory-targeted rehabilitation strategies for low back pain. |
| 10 | References: Up-to-date, but check formatting (e.g., DOI consistency). | Thank you for this important observation. We have carefully reviewed all references for completeness and consistency, specially for DOI consistency. |
| 11 | Writing and Clarity: Minor typos (e.g., "Based on of the included studies" in conclusion; "dppc" vs. "SMD" inconsistencies). Ensure consistent terminology (e.g., "CLBP" vs. "LBP"). | Thank you for highlighting these points. We conducted an extensive revision of the manuscript to improve writing clarity, correct minor typographical errors (e.g., “Based on of the included studies” in the conclusion), and ensure consistent terminology throughout, including the use of “CLBP” versus “LBP” and standardizing effect size reporting as Morris’ dppc rather than SMD. These changes enhance the readability, precision, and consistency of the manuscript. |
| 12 | Supplementary Materials: PRISMA checklist is referenced—verify completion. Include flow diagram (Fig 1) details. | Thank you for this comment. We have carefully reviewed and completed the PRISMA checklist to ensure all items are addressed. Additionally, Figure 1 (PRISMA flow diagram) has been updated to accurately reflect all details now correspond with the information provided in the manuscript and checklist. |
