# Supplementary material for: Effects of breathing exercise and thoracic techniques on pain and disability in low back pain: A systematic review and meta-analysis
Source: PLoS One. 2026 Jan 14;21(1):e0339263. doi: 10.1371/journal.pone.0339263 (PMC12803473; doi:10.1371/journal.pone.0339263)
Supplement: Attachment — Submitted filename: Response_to_Reviewers_auresp_2.docx [file pone.0339263.s006.docx]

Dear respected Editor and reviewers,

We are grateful for the reviewers’ valuable feedback, which has helped us significantly strengthen the scientific rigor and clarity of our manuscript. We believe these revisions further enhance the transparency, methodological robustness, and clinical relevance of the study.

Regards

**Reviewer #1**
*Comment:*
Dear Authors,
I would like to thank you for giving me an opportunity to consider this work for publication. You well done the point-by-point answer to the comments of the reviewers. Thanks.

*Response:*
We sincerely thank the reviewer for their kind words and for taking the time to evaluate our revised manuscript. We greatly appreciate your recognition of our efforts to address all prior comments thoroughly and improve the clarity and quality of the paper.

**Reviewer #2**
*General Comment:*
This revised manuscript shows substantial and meaningful improvement compared with the original version. The authors have carefully addressed most major reviewer comments raised in the first round, particularly those concerning methodological clarity, conceptual depth, and consistency in reporting. The study is now more coherent, transparent, and aligned with PRISMA and GRADE standards.

*Response:*
We thank the reviewer for the positive and constructive feedback. We are pleased that you found the revised version substantially improved in terms of methodological clarity, conceptual depth, and consistency with PRISMA and GRADE standards. We have carefully addressed the remaining concerns as detailed below.

*General Comment:*

The topic remains highly relevant, as it investigates the effects of breathing exercises and thoracic manual techniques—an innovative, non-invasive approach to low back pain (LBP) rehabilitation. The revision strengthens the overall scientific and clinical credibility of the work. However, a few issues related to residual heterogeneity, limited exploration of moderators, and overinterpretation of low-certainty evidence still need refinement before acceptance.

*Response:*
We appreciate the reviewer’s insightful comment. The *Discussion* and *Conclusion* sections have been revised to more clearly address residual heterogeneity, acknowledge potential moderators, and moderate the interpretation of low-certainty evidence. These changes ensure a more balanced and transparent presentation of our findings.

**Specific Comments and Responses**

1. **Comment:** Include abbreviations in table footnotes for clarity (e.g., RMQ = Roland–Morris Questionnaire, ODI = Oswestry Disability Index).
   **Response:** We thank the reviewer for the observation. The abbreviations (e.g., RMQ, ODI, etc.) were already included in the table footnotes in the previous version; we have rechecked and ensured that all abbreviations are clearly defined for consistency and clarity.
2. **Comment:** Ensure figure legends clearly describe the direction of effect and metric (Morris’ dppc).
   **Response:** Figure legends have been revised to explicitly indicate the direction of effect and the metric used (Morris’ dppc).
3. **Comment:** Confirm that all supplementary materials (search syntax, PRISMA checklist, flow diagram) are included and updated.
   **Response:** We confirm that all supplementary materials have been included and updated, including the full search syntax, PRISMA checklist, and revised flow diagram reflecting the final number of included studies.
4. **Comment:** Check reference formatting (consistent DOI presentation).
   **Response:** We have reviewed and corrected all references to ensure consistent DOI formatting according to the journal’s style guidelines.
